# A joint use of pooling and imputation for genotyping SNPs

**DOI:** 10.1186/s12859-022-04974-7

**Published:** 2022-10-13

**Authors:** Camille Clouard, Kristiina Ausmees, Carl Nettelblad

**Affiliations:** Division of Scientific Computing, Department of Information Technology, Uppsala University, Lägerhyddsvägen 1, hus 10, 75237 Uppsala, Sweden

**Keywords:** Pooling, Imputation, Genotyping

## Abstract

**Background:**

Despite continuing technological advances, the cost for large-scale genotyping of a high number of samples can be prohibitive. The purpose of this study is to design a cost-saving strategy for SNP genotyping. We suggest making use of pooling, a group testing technique, to drop the amount of SNP arrays needed. We believe that this will be of the greatest importance for non-model organisms with more limited resources in terms of cost-efficient large-scale chips and high-quality reference genomes, such as application in wildlife monitoring, plant and animal breeding, but it is in essence species-agnostic. The proposed approach consists in grouping and mixing individual DNA samples into pools before testing these pools on bead-chips, such that the number of pools is less than the number of individual samples. We present a statistical estimation algorithm, based on the pooling outcomes, for inferring marker-wise the most likely genotype of every sample in each pool. Finally, we input these estimated genotypes into existing imputation algorithms. We compare the imputation performance from pooled data with the Beagle algorithm, and a local likelihood-aware phasing algorithm closely modeled on MaCH that we implemented.

**Results:**

We conduct simulations based on human data from the *1000 Genomes Project*, to aid comparison with other imputation studies. Based on the simulated data, we find that pooling impacts the genotype frequencies of the directly identifiable markers, without imputation. We also demonstrate how a combinatorial estimation of the genotype probabilities from the pooling design can improve the prediction performance of imputation models. Our algorithm achieves 93% concordance in predicting unassayed markers from pooled data, thus it outperforms the Beagle imputation model which reaches 80% concordance. We observe that the pooling design gives higher concordance for the rare variants than traditional low-density to high-density imputation commonly used for cost-effective genotyping of large cohorts.

**Conclusions:**

We present promising results for combining a pooling scheme for SNP genotyping with computational genotype imputation on human data. These results could find potential applications in any context where the genotyping costs form a limiting factor on the study size, such as in marker-assisted selection in plant breeding.

**Supplementary Information:**

The online version contains supplementary material available at 10.1186/s12859-022-04974-7.

## Background

### Genotyping DNA markers at high density

Biological and medical research e.g. association studies or traits mapping have been interested in Single Nucleotide Polymorphisms (SNPs) genotypes because of their numerous advantages as genetic markers [1]. Among the various tools performing SNP genotyping, the genotyping chips technology (bead-chips) is well-suited for processing many variants at a time.

In association studies, SNPs are used to differentiate subpopulations or individuals from one another when they can be clustered into informative patterns of genetic variation within a sample. Tens or hundreds of thousands of SNPs are often required for achieving relevant, informative, and significant associations or mapping [2]. Despite their abundance, many of the SNPs carrying variation patterns of relevance can be categorized as (extremely) rare variants, e.g. variants with a population frequency less than 1%. Consequently, a large cohort of individuals should be processed to detect these variations and their effects. Computational approaches based on appropriate algorithms offer solutions for increasing both the amount of genotyped markers and the study population size at a reasonable cost. The computational solutions represent a midway to the dilemma of choosing between genotyping a large population at low-density only, or obtaining high-density genotypes sets but for a restricted number of individuals.

A common method to reduce the genotyping cost is to genotype a low-density (LD) set of markers in a study population and to infer a high-density (HD) one. The inference process, which we refer to as classical imputation, is based on a reference population that is assumed to be similar to the study one, and where the genotypes of all markers are known. Imputation methods have demonstrated high accuracy for inferring unassayed genotypes in a population. Nonetheless, several studies found imputation usually performs less well for the rare variants relatively to the common ones [3–7].

### Saving genotyping costs with combinatorial group testing techniques

Pooling is a group testing technique that aims to identify defective samples in a population with the fewest tests possible. Its usage for genetic screening or compressed genotyping was suggested in the 1990s [8]. Numerous studies have proposed the use of pooling for tackling the cost issue for DNA processing [9–11], for instance when conducting DNA variant detection tasks on 96-well PCR-plates. Pooling turns out to be particularly efficient when dealing with the detection of rare variants, as other applications in association studies also show with human [9], animal, and crop data [12, 13]. In this context, the carriers of rare variants are seen as the “defective” items. The applications of DNA pooling in association studies has been mostly used for estimating allelic or haplotype frequencies that are derived from the pooled genotype frequencies. Several papers proposed statistical models that incorporate error-correction mechanisms for taking into account the noisy genotype data from pools. In some cases, the statistic used for testing the allelic association is corrected with the variance of the estimates in the case and the control populations [14–17]. In other cases, the models relies on linear regression models for handling the genotyping errors when estimating the allelic or haplotypic frequencies [18]. More recently, genotype pooling in cattle has been suggested as an avenue for more efficient breeding value estimates in large populations [19].

We propose to implement a similar pooling strategy in order to reduce the cost of SNP genotyping, without sacrificing the power to detect carriers of low-MAF (minor allele frequency) variants or shrinking the study population size. In practice, this is accomplished by pooling samples before them being tested on the SNP chips, with each sample being included in multiple pools. The individual genotypes are then reconstructed based on the test results from the pools. Our study does not target to estimate the overall allelic frequencies at markers, it rather aims to find a large-scale and moderate-cost genotyping method that focuses on the accuracy of every individual genotype estimated.

Various combinatorial group testing schemes have been explored in the literature. These schemes, also called pooling designs or algorithms, can be split into two families, the sequential and the non-adaptive. In the first case, groups (or pools) are consecutively built from the data and tested in several steps whereas in the latter, all groups are constructed and tested at once simultaneously. Since we test all markers on the SNP chip simultaneously in our pooling design, only non-adaptive group testing (NGT) algorithms are suitable for our study [2, 20].

For uniquely identifying and keeping track of every individual contribution to the pool, the designs with overlapping pools were found to be effective and accurate [2, 21–23]. Among the strategies that have been studied for assigning the individuals into overlapping pools, we found mentioned in the literature the DNA Sudoku approach [9] and the Shifted Transversal Design (STD) [24, 25]. Both present a deterministic algorithm for recovering the individual test results from the pools. We have also noted other approaches as compressed sensing [2, 24, 26] which are particularly suitable for processing the rare variants and incorporate probabilities in the decoding step. Our design is a simple case of STD which partitions the samples to be pooled into repeated blocks, where each block corresponds to a pair of layers [20]. Given the characteristics of the pooling design we implement in this study, we designate it by Nonadaptive Overlapping Repeated Block (NORB) design.

When attempting to decode individual genotypes from the pools, some ambiguity may arise, resulting in missing genotype data for some individuals and markers [2, 9]. This drawback is particularly strong when the defective and the non-defective items are in comparable proportions in the population. In our setting where defectives correspond to minor allele carriers at SNPs, this situation is likely to be encountered with the common variants. As suggested by He et al. [23], a likelihood framework can be used for formulating the pooling problem as an extension to the combinatorial methods. The authors found that the likelihood framework and its flexibility is especially suitable for applications that target the accurate genotyping of a population. In this study, we propose to first estimate the likely distribution for each incomplete pooling outcome, and then do a full imputation of all missing genotypes in the data set using more traditional genotype imputation methods.

### Improving pooled genotyping results with imputation methods

Genotype imputation refers to computational approaches for inferring genotypes based on incomplete or uncertain observational data in a population. Many well-performing algorithms for imputation use Hidden Markov Models (HMM) [3, 27] that exploit haplotype-frequency variations and linkage disequilibrium. Other statistical methods such as SNP-tagging based approaches can be found but are not as accurate.

Imputation has been widely used on human genetic data [27–29], but also on plant or animal DNA more recently [30, 31]. To consider pooling and imputation together has been suggested for improving the decoding process performance when genotyping rare variants [10].

On a general level, the imputation problem can be formulated as resolving ambiguous or unknown genotypes with predictions by aggregating population-wide genetic information [3]. Besides the reference population, some imputation methods can incorporate the relatedness between the study individuals, if such data are provided.

We focused on population-based imputation methods, designed for dealing with unrelated individuals. An extensive investigation of the performance-critical parameters that drive imputation is out of the scope of this study, as well as the family-based methods which include pedigree information in the computations. Due to the very common case of very large populations with significant cost constraints in important applications such as animal and plant breeding, we believe that pedigree-aware imputation methods could form an excellent fit with pooling in that context.

Within the population-based methods, two main approaches have been dominating for a long time, namely the tree-based haplotypes clusters and the coalescent models [3, 32]. More recent approaches tend to build on these, but they locally subsample the references based on index searches. We have not included those in this study, since the decoding of pools renders complex patterns of genotype probabilities.

Both population-based models are statistical methods that yield probabilistic predictions for the missing genotypes. They implement HMM based on template haplotypes, but with some differences. In coalescent models, the probabilistic estimation of the genotypes at unassayed markers is computed from a stochastic expectation-maximization (EM) method. Tree-based clustering, implemented in the Beagle software, is an empirical model determined by the counts of similar segments found across the template haplotypes. For both the coalescent and the tree-based models, the hidden states underlying the Markov chain of the HMM are defined by single or aggregated template haplotypes. The way this set of template haplotypes is constituted varies with the imputation method used. The transition from one haplotypic state to another between two consecutive markers mimics a historical recombination event, while the emitted symbols of the HMM are the genotypes, which are modeled as possibly erroneous copies of the hidden pair of haplotypes and hence express mutation events. Depending on the approach, recombination and mutation phenomena are either explicitly parametrized, or captured implicitly.

Among the coalescent models, MACH and IMPUTE2 have been found to perform the best in different studies [27, 29, 33, 34]. We implemented a similar method based on [35] and we refer to this algorithm as *Prophaser* [36] in this paper. To the difference of the common practice in MACH and IMPUTE2, *Prophaser* uses all the available template haplotypes as hidden states in the HMM. All aforementioned methods and software run one HMM for each study individual, and yield probabilistic estimates of the missing genotypes.

IMPUTE2 and MACH form the HMM hidden states by selecting *h* template haplotypes in both the reference and the study population, such there is a constant number $h^2$ hidden states at each of the *j* diploid markers. Hence, these methods have a complexity ${\mathcal {O}}(j h^2)$ in time for each study individual [37], and the time complexity grows linearly as the size of the study population. Despite the use of a memory-saving technique recomputing parts of the forward-backward table on the fly, turning the memory complexity to ${\mathcal {O}}(\sqrt{j}h^2)$, several papers point out computational efficiency issues with MACH [3, 27, 32] when compared to the other methods mentioned. By contrast, Beagle operates a dimension reduction of the hidden states space thanks to its clustering approach, which has been shown to be particularly efficient when imputing large data sets. The successive releases have improved the software performance in this direction [32, 38–41]. In this study, we use Beagle as a comparison baseline for imputation.

### Scope of the study

In this paper, we present a new cost-effective genotyping approach based on the joint use of a pooling strategy followed by imputation processing. We analyze how a pooling procedure, applied on a large data set, impacts what we can conclude about the underlying distribution of genotype frequencies in the study population.

We also evaluate how conventional imputation methods perform when given such a pooled data set which has an unusual and characteristic genotype distribution. Specifically, we investigate if refining the specification of ambiguous genotypes based on the combinatorial outcomes can improve imputation performance. The proposed specific pooling scheme is not unique, however it proves to be a reasonable starting point for evaluating the promise of such designs. Furthermore, we focus solely on the computational aspects of determining genotypes. In practice, proper schemes for performing pooling and SNP genotype quality control would be needed. The resilience of imputation methods to patterns of fully missing markers or fully random genotyping noise is well-known and therefore also not a focus of this study.

## Methods

### Genotyping scenarios

In order to first evaluate how bead-chip genotype data respond to pooling treatment and second, how imputations methods perform on pooled data, we designed the following simulation experiment. We build two marker sets with genotype data from a human population at low respectively high density (LD resp. HD data sets) by extracting only those markers from the *1000 Genomes Project* (1KGP) data set that are present in one lower-density and one higher-density Illumina bead-chip in common use. We then compare the performance of two approaches for genotyping markers at high-density. The first approach serves as a baseline and simulates a usual study case where part of the markers are genotyped at low density in a target population, and the rest of the markers are imputed based on a high-density reference panel. The second approach evaluates genotyping markers at a high density from pools of individuals and then using imputation for those individual genotypes that are not fully decodable from the pooling.

### Data sets and data preparation

We use data from the well-studied reference resource made available by the 1KGP, more specifically phase 3 v5 [21, 29, 42–44], providing genotype data over 2504 unrelated human individuals across 26 subpopulations analyzed worldwide [45].

We select markers from chromosome 20 that has been studied in several previous papers [5, 41, 46]. This chromosome spans approximately 63 million DNA base pairs [42]. Within the 1KGP in the phase 3 version released 2015, 1,739,315 variants are genotyped as biallelic SNPs, out of which 1,589,038 (91.4%) have a minor allele frequency (MAF) less than 5%. These are called rare or low-frequency variants [37, 47].

After selecting the biallelic SNPs, we retain markers that are common to both the 1KGP chromosome 20 data set and analyzed on the Illumina bead-chip products *Infinium OmniExpress-24 Kit* and *Infinium Omni2.5—8 Kit*. Intersecting the markers from the Illumina arrays and the markers genotyped in the 1KGP for the chromosome 20 yields two overlapping experimental maps. The map derived from the OmniExpress bead-chip consists of 17,791 biallelic markers, out of which 17,015 markers are shared with the map derived from the Omni2.5 bead-chip which lists in total 52,697 markers (see Fig. 2a). With respective densities of 1 SNP per 3.5 kb and 1 SNP per 1.19 kb, we hence obtain low-density (LD) and high-density (HD) marker sets [38].

**Fig. 1:**
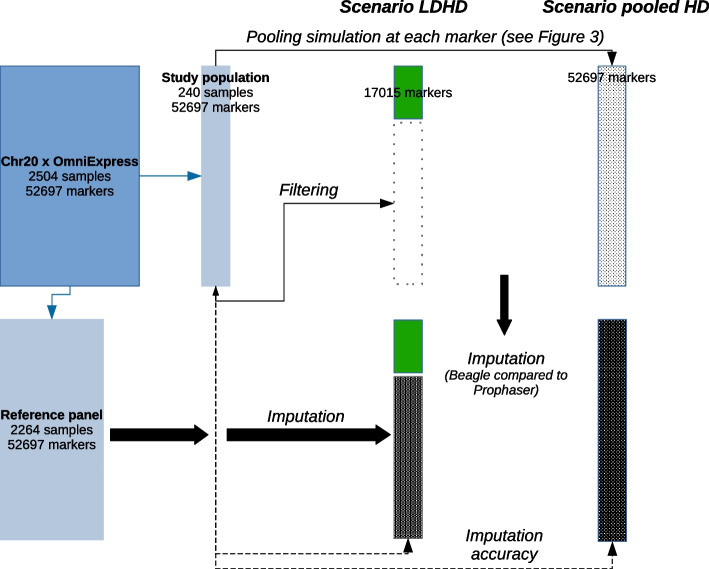
Experimental steps for creating the data sets in the pooling and classical imputation scenarios.The original data set “Chr20 x OmniExpress” consists of the genotype data of 2504 samples at 52,697 SNPs. The set of markers is created by intersecting the variants present on both bead-chips from the Illumina manufacturer and the data for the chromosome 20 in the 1KGP. The original data set is randomly split into a reference panel and a study population. In the LDHD scenario, all markers in the HD data set that are not present in the LD data set are filtered out in the study population. In the pooled HD scenario, the study samples are first assigned to blocks and pools, second the pools are genotyped at all markers in the HD data set, and last the genotype of each sample is decoded from the pools at every marker. See Fig. 3 for an example of the simulation steps in 1 block at 1 marker. The imputation step is performed in both scenarios from the reference panel, with Beagle on the one hand and *Prophaser* on the other hand. The genotyping accuracy in each scenario is computed by comparing the imputed genotypes with the true ones in the original data set for the study population

For simulating imputation, the 2504 unrelated human samples are randomly split into two populations, regardless of their subpopulation. The first one is the reference panel (PNL) with 2264 individuals, the latter is the study population (STU) with 240 individuals, thus observing proportion PNL:STU-sizes of ca. 10:1 as in [3]. For the classical imputation scenario simulation, we delete in the STU population genotype data for the markers only present in the HD data set and keep fully genotyped at LD the 17,015 markers common to both maps. In the pooling scenario, we keep all the 52,697 HD in STU and simulate pooled genotypes as described hereafter. In PNL, we keep the genotype data for all LD and HD markers for both scenarios. Figure 1 gives an overview of the experimental steps carried out in both scenarios.

**Fig. 2:**
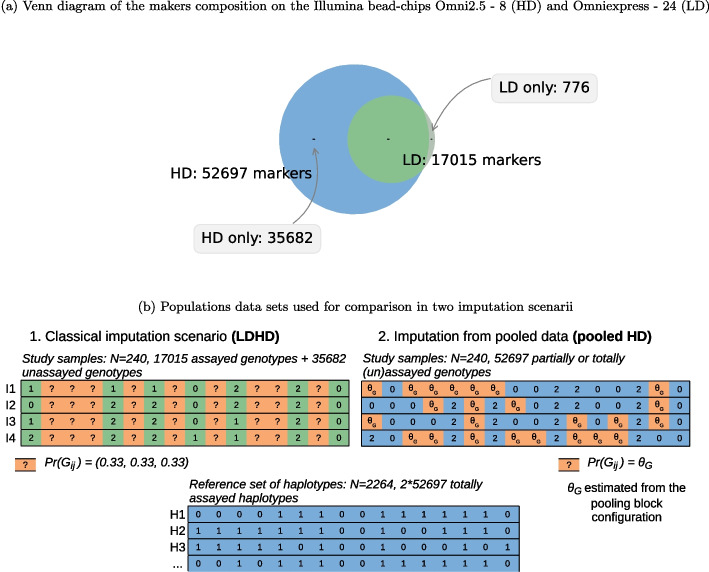
Markers data sets used for the study population in the pooling and classical imputation scenarios. **a** LD and HD markers data sets from intersecting Illumina bead-chips x 1KGP chromosome 20. **b** Missing genotypes repartition and values in a classical imputation scenario (1.), and in an imputation scenario from pooled data (2.) where the genotypes probabilities $\theta _{G}$ are estimated from the configurations of the pooling blocks

Figure 2 illustrates the composition of the different data sets composition before imputation. In both scenarios, after imputation, the study population is eventually fully genotyped at HD markers.

### Group testing design for simulating pooled genotyping from microarrays data

The study population is further processed with pooling simulation, which yields missing genotypes spread in the data.

Based on the DNA Sudoku study [9], we define critical parameters for optimizing the design which are the number of individuals per block, the number of intersecting pools per block holding each pair of samples, and the number of pools that hold any given sample. These parameters and the pooling algorithm can be mathematically formulated as a binary $k \times m$ matrix *M* with *k* rows representing pools and *m* columns representing samples. *M* is called the design matrix of the scheme.

*NORB parameters and design matrix* We choose $n_B = 16$ samples for the block size with pools of degree 4, a samples’ weight equal to 2, and a pool intersection value equal to 1. Hence, we get a number of pools per block equal to 8. The reduction factor $\rho$ is 2, or equivalently the number of individuals is twice the number of pools within a block.

*Square representation of a block* We introduce a graphical representation of a pooling block with genotypes at a given SNP, according to the chosen parameters. As described by Ngo and Du in their taxonomy of nonadaptive pooling designs [25], a simple transversal design can be represented as a grid. The rows and columns $\{P_{t}\}_{1 \le t \le T}$ are the pools, and $\{G_{i}\}_{1 \le i \le n_B} \in \{ -1, 0, 1, 2 \}$ the individuals’ genotypes which is, in order, interpreted as ’missing genotype’, ’homozygous for the reference allele’, ’heterozygous’, ’homozygous for the alternate allele’.

**Table:** 

	P$_{5}$	P$_{6}$	P$_{7}$	P$_{8}$
P$_{1}$	G$_{1}$	G$_{2}$	G$_{3}$	G$_{4}$
P$_{2}$	G$_{5}$	G$_{6}$	G$_{7}$	G$_{8}$
P$_{3}$	G$_{9}$	G$_{10}$	G$_{11}$	G$_{12}$
P$_{4}$	G$_{13}$	G$_{14}$	G$_{15}$	G$_{16}$

Pooling is simulated on the genotypes in the study population (STU data set) for the imputation scenario 2 (pooled HD data). STU was created in view of having a size which is a multiple of the block size chosen, i.e. STU has a size $B_{stu} * n_B = 15*16$, where $B_{stu}$ is the number of pooling blocks formed from the study population. At every SNP, we implemented the pooling simulation as described hereafter.

**Fig. 3:**
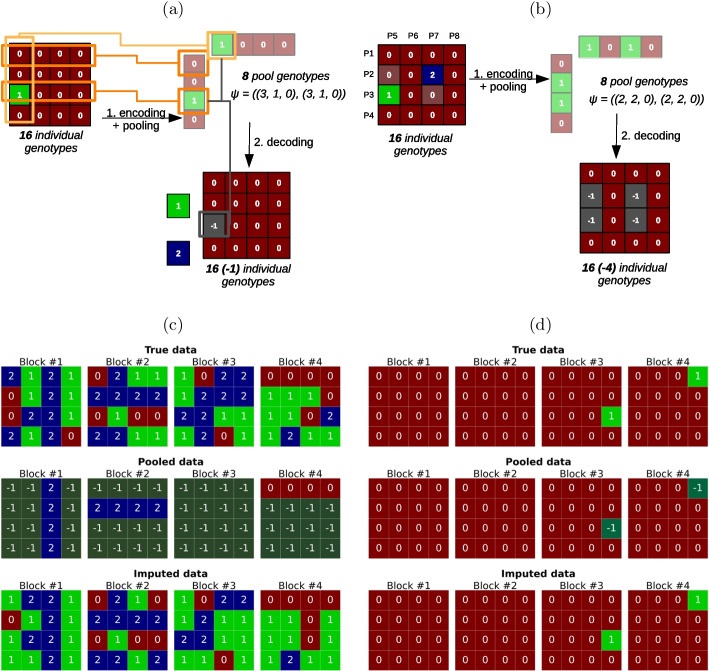
Examples of genotype pooling simulation at the block level. **a** Configuration with 1 sample carrying the minor allele. This carrier is identified after pooling, but not if it has a heterozygous (1) or a minor homozygous (2) genotype. **b** Configuration with 2 samples carrying the minor allele. At least 2 of the 4 samples highlighted in grey are minor allele carriers, but the genotypes of these 4 samples are indeterminate. The first step is encoding and pooling. Encoding assigns every sample to a pool and defines its pool coordinates. For instance in **a**, the sample at the top-left corner of the matrix has coordinates (1, 5). Pooling computes the genotype of a pool as if its would tested on a SNP-chip. Pool 5 (P5, most left) has genotype 1: both alleles 0 and 1 are detected among the samples. Pool 1 has genotype 0 because only the allele 0 is detected. The decoding step infers the pooled genotype of each sample from the genotypes of its coordinates. The genotype can be $-1$ i.e. indeterminate when both coordinates have genotype 1, or fully determined else. In subfigure 3a, the sample with coordinates (3, 5) carries the alternate allele, but there can be 1 or 2 copies of it. $\psi$ is the observed pooling pattern that results from grouped genotyping, given as the number of row- and column-pools having the genotypes (0, 1, 2). In the example 3a, there are 3 row-pools having genotype 0, 1 row-pool having genotype 1 and 0 having genotype 2, likewise for the column-pools. **c** and **d** Simulation example of genotype pooling and imputation outcomes for markers from the 1KGP data (chromosome 20). The genotypes are represented as unphased GT. From top to bottom: true genotype data, pooled genotypes, imputed genotypes. **c** SNP 20:264365, $MAF = 0.4625$. **d** SNP 20:62915126, $MAF = 0.00625$

*Encoding and decoding rules* With the design we have selected for our experiment, simulating pooling on items involves an encoding step followed by a decoding step. Two examples of genotype pooling simulation are shown in Fig. 3a, b.

First, the encoding step simulates the genotype outcome for a pool from the combination of the individual genotypes in it. SNP chip genotyping detects which alleles are present in the sample at each SNP (0 for the reference allele or 1 for the alternate allele) on the chip. That means, in the simulation of the pooling encoding step, a pool has genotype 0 (respectively 2) if and only if all samples forming the pool are homogeneous and have homozygous genotype 0 (resp. 2). Any other intermediate combination of a pool from samples having heterogeneous genotypes 0, 1, or 2 results in a heterozygous pool with genotype 1.

In the second step, decoding the individual genotypes from their intersecting pools is done while assuming there was no genotyping error. In our design, every sample is at the intersection of two pools. If both pools have genotype 0 (or 2), the sample has genotype 0 (or 2). Also, since a pool has a homozygous genotype if and only if all contributing samples have the homozygous genotype, this implies that any individual at the intersection of a homozygous pool and a heterozygous one must be homozygous. In the case of a pooling block with exactly one carrier of the alternate allele (Fig. 3a), if exactly two pools have a heterozygous genotype 1 (pools $P_3$ and $P_5$ in Fig. 3a), we deduce the individual at their intersection has the alternate (or reference) allele, but we cannot state if two copies of this allele are carried (genotype 2, or 0 in the symmetrical case where the reference allele is the minor one) or only one (genotype 1). In this case, ambiguity arises at decoding, in other words, genotype data is reported as missing. To fully assess the probable state of the genotypes of each sample in a pooling block, not only the pools where a sample is included have to be considered but also the full block. We propose to make use of the constraints imposed by the outcome for each pool to estimate the genotype distribution for any undecoded sample. This includes the distribution between heterozygote and homozygote for decoded carriers.

Figure 3c, d show some results we obtain after simulating pooling and imputation at two markers for $4 \times 16 = 64$ samples in the study population: Fig. 3c is an example for a common variant and Fig. 3d illustrates the case of a rarer variant. In practice, genotyping pools of samples on microarrays requires computational processing of the decoding step only.

### Estimation of the genotype probabilities from combinatorial information

At the block level, the pooling scheme implies possible and impossible latent genotypes for a given sample. For example, a decoded block comprising twelve REF-homozygous and four missing genotypes as in Fig. 3b imposes the constraint at least two out of the four samples are minor allele carriers (i.e. genotype in $\{1, 2\}$), whereas the other missing samples can have any genotype in $\{0, 1, 2\}$. Consequently, within these four unknown sample states, the probability of encountering actual homozygous-REF is lower than in a case where the missingness pattern of genotypes is independent of the actual genotype value, as is typically the case in imputation from low to higher density. By proceeding in a similar way for any observable pooling block, we propose to explicitly model the expected distribution of each incompletely decoded genotype.

#### Genotype representations

In this paper, beyond the G representation introduced previously, we use the genotype probabilities (GP) format, which expresses any genotype as a probability simplex over the three zygosity categories. G and GP are equivalent representations, for example if all genotype states are uniformly equally likely to be observed, this results in a genotype probability $GP = (0.33, 0.33, 0.33)$ (i.e. $G=-1$). A determined genotype has one of the following probabilities: $GP = (0, 0, 1)$, (0, 1, 0), or (1, 0, 0) (i.e. $G=2$, $G=1$, or $G=0$).

#### Statistical formulation of the genotype decoding problem

We introduce hereafter the notations and definitions which frame the pooling procedure as a statistical inference problem in missing data. In this framework, we later present an algorithm for estimating the most likely genotype at any missing entry conditioned on the configuration of the pooling block. Our strategy proceeds by enumerating genotype combinations for the missing data that are consistent with the data observed from the pooling blocks, and uses that enumeration to compute an estimate of the latent genotype frequencies.

*Model distribution for the genotypes* Let the genotype *G* be a random variable with three outcomes 0, 1, and 2. The genotype probabilities $\pi$ are expressed as

1 $$\begin{aligned} \pi = ( p_{0}, p_{1}, p_{2} ) \end{aligned}$$

where $( p_0, p_1, p_2 )$ are the probabilities for the genotype 0, 1, and 2 at a given variant for a given sample. Therefore, we model the complete (not pooled) genotype data within a pooling block as an array ${{\textbf {x}}}$ of size $16 \times 3$ ($n_B = 16$) where each data point $x_i$ is a probability simplex $[ p_{0i}, p_{1i}, p_{2i} ]$. Each probability simplex is an indicator vector, since the genotype is fully known.

2 $$\begin{aligned} {{\textbf {x}}}&= ( x_1, x_2, \ldots , x_{16} ) \end{aligned}$$

3 $$\begin{aligned} \forall i \in [1, 16] \qquad x_i&= \begin{bmatrix}p_{0i} \\ p_{1i} \\ p_{2i}\end{bmatrix} \end{aligned}$$

Since the samples are randomly assigned to pooling blocks, the genotype probabilities $x_i$ are independent from each other.

Furthermore, we denote ${{\textbf {z}}}$ the prior probabilities for genotypes that follow pooling and pool decoding. ${{\textbf {z}}}$ is another list of probabilities, where some genotypes are fully decoded, some are fully unrecoverable, and some indicate carrier status, without being able to distinguish between a heterozygous genotype or a homozygous one as on Fig. 3a. The pooled genotypes are represented by

4 $$\begin{aligned} {{\textbf {z}}}&= ( z_1, z_2, \ldots , z_{16} ), \end{aligned}$$

5 $$\begin{aligned} \forall i \in [1, 16] \qquad z_i&= \begin{bmatrix}{\tilde{p}}_{0i} \\ {\tilde{p}}_{1i} \\ {\tilde{p}}_{2i}\end{bmatrix} \end{aligned}$$

The data $z_i$ for each cell of a pooling block is modelled with the simplex of genotype probabilities $( {\tilde{p}}_{0i}, {\tilde{p}}_{1i}, {\tilde{p}}_{2i} )$.

*Mapping of the data space* We denote *layout* the data for the full genotypes ${{\textbf {x}}}$, which is represented as a list of genotype probabilities for each individual in the block. We denote *t* the function transforming ${{\textbf {x}}}$ into ${{\textbf {z}}}$. Since there are several complete layouts ${{\textbf {x}}}$ that could give the same result ${{\textbf {z}}}$ after pooling, *t* is a many-to-one mapping

6 $$\begin{aligned} t :{\mathcal {X}}&\longrightarrow {\mathcal {Z}} \end{aligned}$$

7 $$\begin{aligned} {\mathbf {x}}&\longmapsto {\mathbf {z}} \end{aligned}$$

where ${\mathcal {X}}$ is the space of complete observations, and ${\mathcal {Z}}$ is the space of decoded pooling blocks.

Given the priors $z_i$ for any sample, the problem to solve is to estimate a posterior probability distribution ${\hat{\pi }}_i = ( {\hat{p}}_{0i}, {\hat{p}}_{1i}, {\hat{p}}_{2i} )$ for the three genotypes $\{ 0,1,2 \}$ in any individual, i.e. recovering a probability distribution from which the true genotype $x_i$ can be said to be sampled, as a probabilistic inversion of *t*.

Inherently to the NORB design chosen, the assortment of observable ${{\textbf {z}}}$ is finite and constrained. Moreover, any individual genotype $z_i$ depends on the genotypes of the pools intersecting it, but also on all other pools in the block. Therefore, any sample $z_i$ in the full set of probabilities ${{\textbf {z}}}$ representing the pooling block can be parametrized by the pool configuration and the possible intersections.

*Valid layouts in block patterns* Let $\psi$ be the pooling block pattern described as $\psi = (n_{G_{rows}}, n_{G_{columns}})$, where $n_{G_{rows}}$ (resp. $n_{G_{columns}}$) are the counts of row-pools (resp. column-pools) with encoded genotypes (0, 1, 2). For example, on Fig. 3a, the 8 pools can be described with the block pattern $\psi = ( (3, 1, 0), (3, 1, 0) )$ since there are 3 row-pools having genotype 0, 1 having genotype 1, none having genotype 2, and the same for the column-pools. On Fig. 3b, the pooling pattern is $\psi = ( (2, 2, 0), (2, 2, 0) )$.

We denote ${\mathcal {Z}}_{\psi }$ the space of decoded pooling blocks showing the pattern $\psi$, and correspondingly ${\mathcal {X}}_{\psi }$ the space of the set of valid layouts for $\psi$. A layout is said to be valid with respect to the pattern $\psi$ if applying pooling simulation to ${{\textbf {x}}}$ lets us observe $\psi$ from ${{\textbf {z}}}$. In other words, the valid layouts are

8 $$\begin{aligned} {\mathcal {X}}_{\psi } = \left\{ t_{\psi }({{\textbf {x}}}) \in {\mathcal {Z}}_{\psi }: {{\textbf {x}}} \right\} . \end{aligned}$$

The Additional file 1 shows examples of valid and invalid layouts for the same observed pooling pattern.

*Parametrizing the data mapping* Let $(r, c) \in \{0, 1, 2\}^2$ be the genotype pair of two intersecting pools, such that any $z_i$ is conditioned on (*r*, *c*) . We note that if $(r, c) = (1, 1)$, the decoding of the intersected individual genotype $z_i$ is indeterminate. In other cases, the intersected genotype is fully recoverable as with (0, 1) (resulting in $z_i = [1,0,0]^{\top }$). The pair $(r, c) = (0, 2)$ is not consistent with any genotype, therefore it is never observed.

Based on these notations, we seek to approximate the most likely genotype probabilities $\{ {\hat{\pi }}_{i} \}$ in missing data that are consistent with $x_i$ by using inversion sampling of the priors $z_i$ with respect to $t_{\psi }$. That is to say,

9 $$\begin{aligned} Pr(x_i | \psi ; r, c)&= t_{\psi }^{-1} \big ( Pr(z_i | \psi ; r, c) \big ). \end{aligned}$$

Computing the estimate of the posterior for the missing outcomes as ${\hat{\pi }} := \overline{ {\hat{\pi }}_{i} }$ in a pooling block with pattern $\psi$ by inverse transform sampling is a numerical problem that can be solved as a maximum likelihood estimation (MLE) based on the enumeration of all valid layouts.

#### Maximum likelihood type II estimates

We propose to partition ${\mathcal {Z}}$ into $\{ {\mathcal {Z}}_{\psi } \}_{\psi \in \Psi }$. This enables to marginalize the likelihood over $\psi , r, c$ and lets the problem be solved as a series of separate probability simplex MLE problems in each sample subspace ${\mathcal {Z}}_{\psi }$. The marginal likelihood is sometimes found as type II-likelihood (ML-II) and its maximization (MMLE) as empirical Bayes method. We present as supplementary information a method for computing ${\hat{\pi }}$ by maximizing the marginal likelihood of any observed pattern $\psi$ and deriving genotype posterior probabilities estimates (see Additional file 1). The MMLE example is also well-suited for introducing how we conduct a systematic and comprehensive enumeration of the valid layouts for a given pattern $\psi$.

#### Self-consistent estimations

*Motivation and general mechanism* As a natural extension to the MMLE in presence of incomplete data [48], we implemented a method for estimating the unknown genotypes probabilities inspired by the EM algorithm. The following procedure is applied for each set of parameters $\psi , r, c$.

We initiate the prior estimate of any entry in the block to $z_i = [0.25, 0.5, 0.25]^{\top }$. This choice is based on the assumption that, without information about their frequencies, both alleles at a marker are expected to be equally likely carried.

The algorithm iteratively updates ${\tilde{\pi }} := \overline{ {\tilde{z}}_{i} }$ by alternating between computing the likelihood of the valid layouts using the prior estimate (E step) and deriving the posterior estimate from the frequencies of the genotypes aggregated across the data completions (M step). The M step can incorporate a rescaling operation of the proportions of genotypes that we designate as heterozygotes degeneracy resampling. Eventually, the E and M steps produce a self-consistent estimate ${\hat{\pi }}$ [49] (see Additional file 1 for a calculation example).

Heterozygote degeneracy arises from the internal representation we use for the genotypes under the pooling process. Indeed, the two heterozygous states carrying the phased alleles pairs (0, 1) or (1, 0) are collapsed into a single heterozygous genotype $GP = ( 0, 1, 0 )$ (or equivalently $G=1$). In a way analogous to for example the particles paths in particles filter models, we define this collapsing as heterozygous degeneracy. For instance, a layout involving 4 heterozygous genotypes should be subdivided into $2^4$ micro layouts combining alleles pairs (0, 1) and (1, 0). More generally, the heterozygous degeneracy has order $2^{n_1}$, where $n_1$ is the number of items having genotype 1 in the layout. In practice, enumerating these micro layouts would increase the computation time a lot. Instead, we include the higher probability for heterozygotes internally in the model, taking the degeneracy into account when normalizing, and again when producing the final likelihoods to be used in the imputation process, where a uniform distribution is the expected structure for data without any informative prior.

*Equations of the optimization problem* We proceed in a way identical to MMLE for enumerating all possible completions for the $n_m$ unknown genotypes. At each iteration *m*, The E step calculates first the marginal likelihood of every layout by sampling its genotypes from ${\tilde{\pi }}^{(m-1)} | \psi$. The mixing proportion ${\mathbb {E}} [ {{\textbf {x}}} | {{\textbf {z}}}, {\tilde{\pi }}, \psi ]^{(m)}$ of each layout is computed from all aggregated likelihoods and for any ${{\textbf {z}}} \in {\mathcal {Z}}_{\psi }$. A breakdown of the formula for ${\mathbb {E}} [ {{\textbf {x}}} | {{\textbf {z}}}, {\tilde{\pi }}, \psi ]^{(m)}$ is provided in the Additional file 1.

The M step recomputes the genotype frequencies $( {\tilde{p}}_0, {\tilde{p}}_1, {\tilde{p}}_2 )$ by applying MLE to the likelihoods calculated at the E step.

10 $$\begin{aligned} \tilde{p_{k}}^{(m)}&= \frac{ \sum \limits _{{{\textbf {x}}} \subset {\mathcal {X}}} n_k \, {\mathbb {E}} [ {{\textbf {x}}} | {{\textbf {z}}}, {\tilde{\pi }}, \psi ]^{(m)} }{ \sum \limits _{k} \sum \limits _{{{\textbf {x}}} \subset {\mathcal {X}}} n_k \, {\mathbb {E}} [ {{\textbf {x}}} | {{\textbf {z}}}; {\tilde{\pi }}, \psi ]^{(m)} }, \end{aligned}$$

11 $$\begin{aligned}&k \in \{0, 1, 2\} \end{aligned}$$

where $n_k$ is the counts of genotype *k* observed in the layout ${{\textbf {x}}}$.

Since we do not compute the distribution of the genotype frequencies from the allelic dosage, we suggest a resampling step after the M step that artificially accounts for the heterozygous degeneracy. Hence, we introduce arbitrary weights $w = ( w_0, w_1, w_2 ) = ( 1,2,1 )$ for rescaling $( {\tilde{p}}_0,{\tilde{p}}_1, {\tilde{p}}_2 )$. If we do not account for the heterozygote degeneracy, we pick these weights as $w = ( 1,1,1 )$.

12 $$\begin{aligned} {\tilde{p}}_{k}^{(m) \prime }&= \frac{ w_k \, {\tilde{p}}_{k}^{(m)} }{ {\tilde{p}}_{k}^{(m-1)} }, \ k \in \{0, 1, 2\} \end{aligned}$$

13 $$\begin{aligned} {\tilde{p}}_{k}^{(m) \prime \prime }&= \frac{ {\tilde{p}}_{k}^{(m) \prime } }{ \sum \limits _{k} {\tilde{p}}_{k}^{(m) \prime } } \end{aligned}$$

14 $$\begin{aligned} {\tilde{\pi }}^{(m)}&= ( {\tilde{p}}_{0}^{(m) \prime \prime }, {\tilde{p}}_{1}^{(m) \prime \prime }, {\tilde{p}}_{2}^{(m) \prime \prime } ). \end{aligned}$$

At the last iteration, when the algorithm has converged, the final estimate of ${\tilde{\pi }}$ is computed from a modified version of rescaling, where we compensate for the artificial upscaling used in the previous steps

15 $$\begin{aligned} \hat{p_k}^{(m)} | \psi&= \frac{ (1/w_k) \, {\tilde{p}}_{k}^{(m)} }{ \sum \limits _{k} (1/w_k) \, {\tilde{p}}_{k}^{(m)} }, \ k \in \{0, 1, 2\} \end{aligned}$$

16 $$\begin{aligned}&{\hat{\pi }} | \psi = ( \hat{p_0}^{(m)}, \hat{p_1}^{(m)}, \hat{p_2}^{(m)} ) \end{aligned}$$

Such self-consistent iterative methods provide local distribution estimates for the undecoded genotypes at the pooling block level, based on information from the pooling design. They are independent of the overall MAF in the population because of the choice we made for the prior, and do not take into account the genetic variations specific to the population and its structural traits.

### Imputation for retrieving missing genotypes

For each sample in the study population, we use the aforementioned estimated genotype probabilities ${\tilde{\pi }} | \psi , r, c$ as prior beliefs $\theta _G$ in imputation. Figure 2 summarizes the experimental settings for both this scenario and the classical one. We compare the imputation performance on pooled SNP genotype data of two population-based algorithms, representing each the haplotype clustering approach and the coalescence principle.

#### A haplotype clustering method: Beagle

In this work, Beagle is used in its 4.0 version and with the recommended default parameters. This software version is the best performing release having the features needed for this study. Beagle 5.0 is available but this version does not support logged-GP (GL) data type as input.

We use the HapMap GRCh37 genetic map suggested by Beagle developers and consistent with the genome assembly underlying the version of the 1KGP data used [38]. In practice though, we have not noticed clear deterioration when conducting imputation on pooled data without providing any genetic map.

For the classical imputation scenario, we beforehand verify equivalent results and performance are obtained both if Beagle is run on genotypes in a GT format or GL format. In the first case, unassayed HD markers were set to ./. and in the latter, to $(-0.481, -0.481, -0.481)$. As advised in the documentation, we imputed the entire STU population in the same batch.

In the pooling scenario, we used the same reference panel, but we deliberately chose to run Beagle sample-wise for avoiding the very specific genetic structure of pooled data being used as template haplotypes. Preliminary testing showed a clear deterioration in results if this was not done.

#### A coalescence-based method for haplotype phasing and imputation: prophaser

The original version of MACH did not support GL as input study data, in contrast to IMPUTE2. The main motivation for writing the *Prophaser* [36] code was to implement this feature with full control of e.g. cutoff thresholds for close-to-zero probabilities. The reference panel is read from GT data.

*Prophaser* phases and imputes unassayed markers sample-wise and independently from the rest of STU. Whereas MACH and IMPUTE2 include strategies for selecting a subset of reference samples for computational efficiency reasons, we decided to consistently use the full reference panel as templates in a single iteration estimation. Hence, *Prophaser* uses all reference haplotypes as templates.

### Evaluation of the experimental design

We quantified the performance of the two genotyping scenarios with the concordance rate and cross-entropy. In both cases, the original data from 1KGP in the study population were used as the ground truth, and the predicted data were the imputed genotypes in the same study population.

*Concordance* The most widely used imputation quality metric is the genotype concordance measure which counts the number of matches between the true and the best-guess imputed genotypes. A homozygous genotype imputed as heterozygote (or conversely) is counted as a half mismatch, and a homozygote imputed to its opposite homozygote as a full mismatch. Concordance sometimes appears as its complementary formulation with the discordance rate [3]. Several publications refer to the concordance rate directly as the genotype accuracy rate [39] or as imputation accuracy [32], whilst the discordance rate is designated as the imputation error rate [33, 38].

*Cross-entropy* In the studies presenting the successive Beagle software versions, the accuracy in the sense of the concordance does not quantify how similar the imputed genotypes are to the true ones. This has already been pointed out by e.g. Nothnagel et al. [27]. As an example, we can consider the two following cases: *(a)* a true genotype $G = 1$ being imputed with $GP = ( 0.56, 0.42, 0.02 )$, and *(b)* a genotype $G = 1$ being imputed with $GP = ( 0.7, 0.28, 0.02 )$. Using the best-guess genotype definition, both genotypes will be imputed as $G = 0$ and hence a discordance of one point, but the prediction *(a)* is “weaker” since it has a lower best-guess likelihood ($0.56 < 0.7$). In that sense, the prediction *(a)* should be considered as less significant than the *(b)* one even if both are wrong. Therefore, we introduce the cross-entropy metrics $\chi$ as a divergence measure of the predicted genotype distribution. The cross-entropy we propose is defined as in equation 17 at the *j*-th marker for N individuals imputed.

17 $$\begin{aligned} \chi _j = \frac{ \sum \limits _{i=1}^{N} \left( - \sum \limits _{g=0}^{2} Pr(G_{ij}=g) \ log({\mathcal {L}}_{ijg}) \right) }{ N } \end{aligned}$$

where ${\mathcal {L}}_{ijg}$ is the genotype likelihood (or posterior imputed genotype probability) for the genotype state *g* at the *j*-th marker for the *i*-th individual. For low-probability genotypes, we used a cut-off of $log(10^{-5})$ if the genotype probability was less than $10^{-5}$.

### Computational tools

Due to their computational costs, imputation algorithms were run on compute servers. The computing resources were provided by SNIC through Uppsala Multidisciplinary Center for Advanced Computational Science. This infrastructure provides nodes (compute servers) of two 10-core Xeon E5-2630 V4 or two 8-core Xeon E5-2660 processors running at 2.2 GHz, with 128 to 512 GB memory.

## Results

### Genotype distribution before imputation

**Table 1:** Markers counts and proportions on the LD and the HD maps per MAF bin

MAF	0.00–0.02	0.02–0.04	0.04–0.06	0.06–0.10	0.10–0.20	0.20–0.40	0.40–0.50	Total
LD map (counts)	520	779	673	1537	3969	6561	2976	17015
HD map (counts)	12775	5235	2823	4766	9009	12613	5476	52697
LD map (%)	0.987	1.478	1.277	2.917	7.532	12.450	5.647	32.288
HD map (%)	24.242	9.934	5.357	9.044	17.096	23.935	10.392	100

The counts are given in the two first rows of the table, the proportions in the two last ones. The proportions are given relatively to the total number of SNPs on the HD map. The HD map is on the whole 3 times denser than the LD map but the density is not uniformly increased over the MAF bins. Almost 25% of the markers on the HD map are very rare variants ($MAF < 0.02$), that is 25 times denser than on the LD map where they represent less than 1% of the markers

The LD and HD marker sets built for the experiment both contain SNPs in the whole allelic frequency range but the markers are unevenly distributed over this range. Table 1 provides further details about the uneven distribution. We aim to analyze the uncommon variants at a finer scale and visualize their joint response to pooling and imputation. Therefore, the bins chosen are tighter towards the least MAF values and the boundaries set to [0.0, 0.02, 0.04, 0.06, 0.1, 0.2, 0.4, 0.5] for the intervals.

The most rare variants $(MAF<2\%)$ represent a substantial share of the studied SNPs with 520 markers in the LD dataset and 12,775 in the HD dataset. One should note that even denser chips, or the full marker set of called SNPs in the 1KGP dataset, are even more extreme in this regard.

**Table 2:** Exact genotypes in markers per data MAF bin

MAF	0.00–0.02	0.02–0.04	0.04–0.06	0.06–0.10	0.10–0.20	0.20–0.40	0.40–0.50
*Scenario: LD + HD*							
Number before imputation	520.000	779.000	673.000	1537.000	3969.000	6561.000	2976.000
*Number after imputation*							
Beagle	12699.362	5167.613	2776.687	4673.658	8804.892	12301.371	5337.921
Prophaser	12727.142	5193.438	2793.221	4705.346	8870.104	12396.258	5379.408
Proportion before imputation	0.041	0.149	0.238	0.322	0.441	0.520	0.543
*Proportion after imputation*							
Beagle	0.994	0.987	0.984	0.981	0.977	0.975	0.975
Prophaser	**0.996**	0.992	0.989	0.987	0.985	0.983	0.982
*Scenario: pooled HD*							
Number before imputation	12534.608	4826.542	2396.671	3481.896	4249.592	1853.529	159.575
*Number after imputation*							
Beagle	12565.650	4892.246	2478.292	3778.296	5637.525	5407.479	1941.162
Prophaser	12755.854	5184.621	2758.079	4532.467	7964.742	9858.467	4012.725
Proportion before imputation	0.981	0.922	0.849	0.731	0.472	0.147	0.029
*Proportion after imputation*							
Beagle	0.984	0.935	0.878	0.793	0.626	0.429	0.354
Prophaser	**0.999**	0.990	0.977	0.951	0.884	0.782	0.733

The number of markers is given as the average over all samples in the study population per bin. The proportion of markers is given relatively to the number of markers per bin. To the difference of concordance, only full matches with the true genotype are counted, not half-matches. For the LD + HD scenario, the number of exact genotypes before imputation is equal to the number of variants on the LD map. For the pooled HD scenario, the number of exact genotypes before imputation is equal to the average number of genotypes that are fully determined after pooling simulation. Simulating pooling followed by imputation with Prophaser yields a gain in accuracy for the very rare variants ($MAF < 0.02$) which are almost all exactly genotyped. This gain is not negligible given the low occurence of these variations

The best accuracy scores achieved by *Prophaser* are marked in bold

Table 2 shows the proportion of assayed and determined genotypes before imputation in the LDHD scenario and in the pooled HD scenario.

Already at the preimputation stage, the pooling mechanism proves to be particularly efficient for capturing the most rare variants ($MAF < 2\%$) with 98.1% determined genotypes before imputation. In the LDHD scenario, only 0.41% of the genotypes are assayed in the most rare variants before imputation. In total, there are 67.7% unassayed genotypes before imputation in the LDHD scenario and 44% in the pooled HD scenario. The proportions of known genotypes however varies depending on the MAF.

Whilst the proportion of known genotypes seems to augment as the MAF increases in the LDHD scenario, a negative correlation between the known data rate and the MAF is noticed in the pooling case. Indeed, the proportion of fully decoded genotypes is less than 10% for MAF exceeding 30%. Such markers are common variants. Since both alleles have roughly the same frequency in the population, heterozygotes and mixed genotypes within pools will be far more common as on Fig. 3b, or with even more carriers of the minor allele in the block. To summarize, there is a significant correlation between true genotypes and the probability of the genotype being decoded, and that correlation is further dependent on the MAF of the marker. The proportions of known genotypes before imputation per MAF-bin in the LDHD scenario is actually fixed by the choice made for the LD map. In other words, changing the LD map will modify the distribution of known markers. In the pooled HD scenario, the proportions mostly depend on the MAF of every marker and the HD map chosen has a limited impact on the distributions of known markers per MAF-bin.

**Fig. 4:**
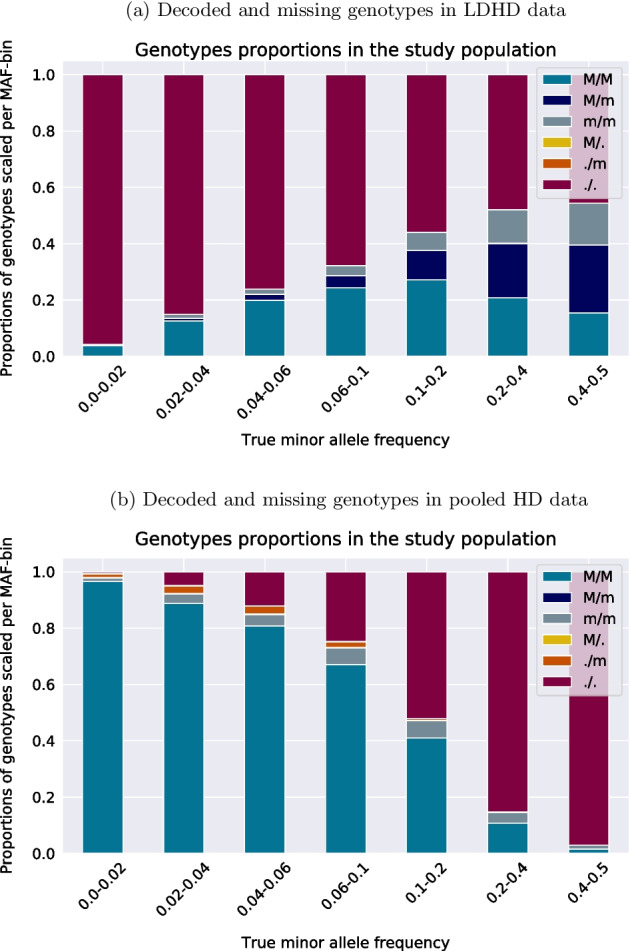
Decoded and missing genotypes in data for both imputation scenarios.The minor and major alleles are denoted *m* and *M*. For simplicity, the simulated decoded genotypes from pooling are represented in GT format. We remind adaptive GL are provided later in the experiment for running imputation on data informed with the pooling outcomes. Half-decoded (GT = *M*/. or ./*m*) and not decoded (GT = ./.) genotypes are considered as missing data. The relative genotypes proportions are scaled in [0, 1] within each bin. **a** The markers only in the LD data set are fully assayed, all other markers have been deleted. **b** True heterozygous genotypes (dark blue) are never fully decoded, whereas the rare variants are almost all fully decoded or at least one of the two alleles is determined

The distribution of heterozygous and homozygous genotypes obtained in each MAF-bin from both data deletion (LDHD scenario) and pooling simulation (pooled HD scenario) are presented on Fig. 4. To the difference of the LDHD data set, the pooled HD one let some markers being half-genotyped in that sense one out of the two alleles can be determined before imputation. For example in the markers having a MAF less than 2%, in addition to the large share of exactly determined genotypes ($GT = M/M$), most of the indeterminate genotypes are yet half-known ($GT = ./m$). The pooling process never fully decodes the true heterozygous genotypes, hence the proportion of unassayed genotypes will be large in common markers. Only the homozygous genotypes can be determined from pooling with our design. For the LDHD scenario, the heterozygous genotypes that are naturally present in the study population at the markers on the LD map are observed in the preimputation data set. These observations highlight the very different compositions of the LDHD and the pooled HD data sets before imputation. On the whole, the distribution of the observed and assayed genotypes in the population is unevenly affected by pooling and depends on the MAF.

### Genotyping accuracy after imputation

**Table 3:** Proportion of exact genotypes after imputation for indeterminate data in the pooled HD scenario per data MAF bin

MAF	0.00–0.02	0.02–0.04	0.04–0.06	0.06–0.10	0.10–0.20	0.20–0.40	0.40–0.50
Prophaser	**0**.**932700**	0.886214	0.849634	0.820339	0.783430	0.745528	0.724745
Beagle	0.124773	0.156686	0.187206	0.227121	0.287044	0.329487	0.334919

This table focuses on the genotypes that are indeterminate after the pooling simulation. The proportion is calculated for these markers only and relatively to the number of markers in the bin. For the very rare variants ($MAF < 0.02$), the indeterminate genotypes are the rare allele carriers. Phaser succeeds in imputing exactly most of them from the provided prior genotype probabilities estimates

The best accuracy scores achieved by *Prophaser* are marked in bold

Table 2 also shows the proportion of genotypes that are imputed exactly to the true one. Table 3 provides a closer insight into the imputation performance of Beagle and *Prophaser* in terms of exact matches for the genotypes at undecoded markers only in the pooled HD scenario.

**Fig. 5:**
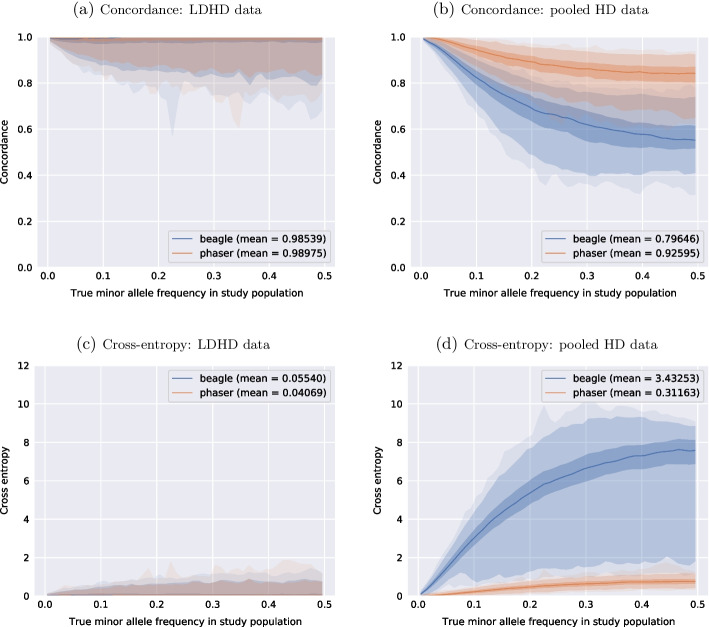
Genotypes imputation accuracy in a classical and a pooled scenario. **a** and **b** concordance (based on best-guess genotype) **c** and **d** cross-entropy (based on posterior genotypes probability) metrics. All markers from the HD map have been used for computing the metrics (52,697 markers). Beagle (labeled as “beagle”) performance is in blue, and Prophaser (labeled as “phaser”) in orange. The central line is the median and the shadowed areas delimit the percentiles 0.0, 0.01, 0.25, 0.75, 0.99, 1.0. The x-axis was built from 0.05-long MAF bins within which each marker concordance score was computed as the mean score of the 500 previous and 500 next markers sorted per ascending MAF

Figure 5 presents the genotyping accuracy for imputed markers in both the LDHD and the pooled HD scenarios. The concordance and cross-entropy metrics are presented for comparison. Preliminary experiments (unpublished results) showed that the strategy of using pooling patterns-adapted GL values instead of uninformed ones improves the imputation accuracy.

In the LDHD scenario, Beagle shows as expected very good performance with an average concordance of 98.5% and low entropy (0.05). The performance is stable across the MAF range on average, though there is a larger variation in accuracy for more common variants. In the pooled HD scenario, while the overall proportion of missing data is lower, Beagle’s performance drops substantially (79.6% concordance on average and a cross-entropy score of 3.43). The wide envelope for the cross-entropy also indicates that the amplitude of prediction errors on the marker level varies widely in the pooled HD scenario. The haplotype-clustering model seems to struggle with the unusual genetic structure of pooled data.

*Prophaser* achieves higher accuracy than Beagle in the LDHD scenario, showing nearly 99% average concordance and 0.04 cross-entropy score. As for Beagle, the concordance is stable but more spread for higher MAF (less accurate). In the pooled HD scenario, *Prophaser* clearly outperforms Beagle for imputing the undecoded genotypes by maintaining an average concordance of 92.6% and a cross-entropy score of 0.31. The quantile envelopes for both metrics demonstrate that *Prophaser* gives stable performance for most markers, while the results for Beagle show a much greater variation. It is naturally important not only that the average concordance or entropy is good, but that any single imputed marker of possible importance is trustworthy. Despite the weaker performance on the pooled HD data compared to the LDHD scenario, *Prophaser* proves the ability to use the uncertain decoded genotypes from pooling for successful imputation.

Table 2 gives a detailed view of the number and proportions per MAF bin of exact genotypes, both in the LDHD and in the pooled HD data sets, before and after imputation. It reveals the benefit that is obtained from gneotyping pooled samples for the variants having a MAF less than 2%. *Prophaser* indeed succeeds in raising the proportion of exactly matched genotypes after imputation by 0.3%. This gain is not negligible given the very low frequency of the variations in such markers.

### Computational performance

For Beagle, the compute server (node) was two 10-core processors running at 2.2 GHz with 128 GB memory. For *Prophaser* the node resources were two 8-core processors running at 2.2 GHz, with 128 GB memory. Computation times per study sample were about 7 min for Beagle respectively 6 h 40 min for *Prophaser*, and the memory requirements for each sample consumed about 2.2 GiB (resp. 35 GiB) of memory. In the classical scenario, it is even possible to run Beagle on all study samples together in about 20 min using ca. 12 GiB memory and to get the same accuracy results. Hence, accordingly to the results found in other studies, Beagle demonstrates an excellent computational efficiency in imputing large data sets. *Prophaser* is on the contrary computationally very expensive, as mentioned to be a drawback in the literature with similar algorithms. However, we have not yet optimized the performance of our implementation.

## Discussion

As we could expect, pooling enables efficient identification of carriers of rare variants within the population, but yields high missing data rates for more common variants. Several studies have indeed shown that the distribution of the undecoded items is hypergeometrical and correlated to the minor allele frequency [2, 11]. In the case of low-MAF SNPs, the pools are mostly homogeneous and homozygous, or contain at most one rare variant carrier as on Fig. 3a. Blocks as on Fig. 3b are unlikely to be observed for these SNPs. Indeed, with respect to HWE in a random mating population, rare variant carriers would almost exclusively be heterozygotes. The pooling design used in this study guarantees a theoretical perfect decodability of the samples genotype if at most one sample in the block is carrying the minor allele ($d_0 = 1$, calculated as in the DNA Sudoku [9]). The results presented in Table 2 comply with the theoretical limiting decoding power. The upper bound for MAF with high certainty of decodability is calculated as $\delta _{MAF} = \frac{d_0 \times G_1}{2 \times n_B} = \frac{1 \times 1}{2 \times 16} \approx 3.1\%$. Our results for the pooled HD scenario show that the number of known markers before imputation drops when the MAF is larger than 2%, and decreases even more when the MAF is greater then 4%. SNPs having a MAF below this boundary of 3.1% are expected to be nearly fully assayed in the study population or decoded as rare variant carriers, such that pooling provides a useful complementary process to imputation for achieving accurate genotyping of rare variants that are usually more difficult to impute. Other pooling designs can be explored for increasing the decoding power. With a given pooling design, hybrid procedures consisting of imputation from a fully a assayed LD set and a pooled HD set are further alternatives to consider. Similarly to the representation [22] suggested for evaluating the pooling design performance for clone-based haplotyping, we think that quantifying the genotyping effort in relation to the decoding rate and to the MAF as a performance ratio of pooled genotyping could be a future criterion for choosing a pooling design depending on the markers data set and its characteristics. Considering the very good performance of imputation in a LDHD scenario and the complementary nature of a pooled scenario that excel at capturing the rare variants, one could also imagine a more sparse pooling scheme, such as a $5\times 5$ design, with a dense chip, augmented by full LD testing of some or all individuals. This would give the imputation process a clear scaffold to start out from, together with very accurate information for carriers of rare variants. It also opens perspectives for genotyping on even denser chips targeting very rare variants ($MAF < 0.02$) without large increase in laboratory costs.

We have presented algorithms that locally adapt the genotype frequencies to every pooling block, but we believe further research could be conducted for improving the GL estimates. In our context, the resulting probabilities after decoding should be evaluated in terms of to what extent they improve the imputation results.

Imputation on pooled data yielded notably different performance depending on the imputation method family used. The clustering model as implemented in Beagle seems to suffer from the pooled structure in the data. We think the clusters built collapse together haplotypes that are substantially different, but can have superficial similarities after the decoding of pooled data. This fact also results in the decoded population looking systematically different from the reference population. We showed with the *Prophaser* algorithm that the coalescence assumption supports an imputation model that delivers high accuracy in pooled genotype reconstruction, at a computational cost. This is consistent with other studies [29, 50] that have found the coalescent methods to be robust towards unknown genetic population structures. From the perspective of the method, the systematic bias introduced by the decoding is similar to unknown population structure. By using all the reference haplotypes from the panel during imputation, *Prophaser* might overcome the pitfall of sensitivity to deviant genetic structure as mentioned in [3]. As a result, allele frequencies assessed in the study population are no longer consistent with the effective frequencies differences expressing genetic variation found in the reference panel. While the reason presented in that paper is chip quality, we face similar biased structural heterogeneity issues with pooled data.

This initial investigation of the performance of pooling and imputation as a combined way to recover genotypes is purely based on simulations, in the absence of genotyping errors. In quality control data from chip manufacturers, detection power for alleles can be found on a per-SNP level. Actual detection performance could be influenced by the amount of DNA contributed from various samples within a pool. Our intention is to continue to explore our approach on actual assays, in partnerships where cost-effective genotyping on a massive scale is a real concern.

It should be noted that our probabilistic decoding method could be modified to account for genotyping errors, and that it will be crucial to consider the overall effect of errors in decoding individual SNPs and how those errors in turn affect the ability of the imputation methods to properly reconstruct the haplotype mosaic, since it is the accuracy of that mosaic of reference haplotypes that in turn will influence imputation performance.

## Conclusions

The findings of this study suggest that pooling can be jointly used with imputation methods for achieving accurate SNPs at high density while reducing the actual number of genotyping procedures done on microarrays. However, the atypical structure introduced by pooling in the genotype data requires specific attention and processing for ensuring the best imputation performance possible.

Overall, pooling impacts the allelic and genotypic distributions, and introduces a specific structure in the genetic data which does not reflect their natural distribution. We have described a statistical framework that formalizes pooling as a mathematical transformation of the genotype data, and we have proposed in this framework an algorithm for estimating the latent values of undecoded genotypes. Lastly, thanks to a simulation on real human data, we have shown that a coalescence-based imputation method performs well on pooled data, and that informing imputation with estimates of the latent missing genotypes improves the prediction accuracy. We also presented an implementation (*Prophaser*) of this imputation method for pooled genotype data. Overall, this study provides a first prototype for the computational aspect of a SNP genotyping strategy at a reduced cost by halving the number of microarrays needed compared to a full sample-wise genotyping.

## Supplementary Information

**Additional file 1.** Estimating genotype probabilities in pooled blocks with marginal likelihoods, self-consistency and heterozygotes degeneracy.

## Data Availability

The dataset(s) supporting the conclusions of this article (are) available in the data subdirectory of *genotypooler* repository, https://github.com/camcl/genotypooler/data. These datasets are created from publicly available 1000 Genomes dataset [45]. *Prophaser* code can be found at https://github.com/kausmees/prophaser.

## Abbreviations

1KGP: 1000 Genomes Project
AAF: Alternate allele frequency
EM: Expectation-maximization
GL: Genotype likelihood
GP: Genotype probability
GT: True genotype
HD: High density
HMM: Hidden Markov models
LD: Low density
MAF: Minor allele frequency
MLE: Maximum likelihood estimation
ML-II: type II likelihood, marginal likelihood
MMLE: Maximum marginal likelihood estimation
NGT: Nonadaptive group testing
NORB: Nonadaptive overlapping repeated block
PNL: Reference panel
SNP: Single nucleotide polymorphism
STD: Shifted transversal design
STU: Study population
