# Supplementary material for: A joint use of pooling and imputation for genotyping SNPs
Source: BMC Bioinformatics. 2022 Oct 13;23:421. doi: 10.1186/s12859-022-04974-7 (PMC9563787; doi:10.1186/s12859-022-04974-7)
Supplement: Supplementary file 1 — Additional file 1. Estimating genotype probabilities in pooled blocks with marginal likelihoods, self-consistency and heterozygotes degeneracy. [file 12859_2022_4974_MOESM1_ESM.pdf]

Estimating genotype probabilities in pooling blocks

Camille Clouard[1]

[1]Department of Information Technology, Division of Scientific Computing, Uppsala University

December 1, 2021

The marginal likelihood maximization and the self-consistent procedures, based on the Expectation-Maximization (EM) method, can be implemented for computing genotype probabilities at undecoded items in pooled blocks. The pooling design used is largely based on the DNA Sudoku [1], with a pool size of 4 and a weight of 2. We assume the genotype data we use come from biallelic SNPs of a diploid organism.
The examples presented hereafter apply to a single pooling block of $n_B = 16$ samples, for which we describe different related versions of our genotype likelihood maximization algorithm.

Notations

Pooling transformation of the complete into incomplete genotype data

A genotype is modeled as discrete variable $G \in \{0, 1, 2\}$. Let us define the pooled genotypes $\mathbf{z}$ as a transformed vector from the true genotype vector $\mathbf{x}$ through the pooling function t

$$t\colon \mathcal{X} \longrightarrow \mathcal{Z} \tag{1}$$
$$\mathbf{x} \longmapsto \mathbf{z} \tag{2}$$

where $x = (x_1, x_2, ..., x_n)$ and $z = (z_1, z_2, ..., z_n)$. The genotype vector $\mathbf{z}$ is incomplete in the sense some genotypes might be missing after pooling, these are considered as latent variables. $\mathbf{x}$ is a complete realization of G whereas the missing entries in $\mathbf{z}$ are indicated with the -1 value.

Let us define the observed genotypes configuration (or pattern) in a pooled block as $\psi = (G_{n_{rows}}, G_{n_{columns}})$, where $G_{n_{rows}}$ (resp. $G_{n_{columns}}$) are the counts of row-pools (resp. column-pools) having, in this order, the genotypes $(0, 1, 2)$. For example, the pattern $\psi = ((2, 2, 0), (2, 2, 0))$ represents a block where 2 row-pools have the genotype 0, 2 row-pools have the genotype 1, and none has genotype 2. The column-pools are distributed likewise (see Figure 1 (a)).

Valid data completions from the pooling patterns

The objective is to find the subvectors of ordered genotypes that complete $\mathbf{z}$ to $\mathbf{x}$. In the previous example of pooling pattern $\psi = ((2, 2, 0), (2, 2, 0))$, there are several corresponding vectors $\mathbf{z}$ that subsume to the configuration constraint. The square-block representations of these row-major ordered vectors have in common the 4 missing genotypes are placed on 2 different rows and 2 different columns. For instance, both $\mathbf{z} = (0, 0, 0, 0, -1, 0, -1, 0, -1, 0, -1, 0, 0, 0, 0, 0)$ and $\mathbf{z} = (0, 0, -1, -1, 0, 0, -1, -1, 0, 0, 0, 0, 0, 0, 0, 0)$ are ψ layouts (see the two first block examples on the Figure 1 (a). In this case, there are actually $\binom{4}{2} \times \binom{4}{2} = 36$ vectors $\mathbf{z}$ underlying $\psi = ((2, 2, 0), (2, 2, 0))$, while there are in total $\binom{16}{4} = 1820$ vectors of 16 samples with 4 missing items.

There are 12 distinct permutations from this set, out of which only 4 are valid layouts. The ordered vector $(0, 1, 2, 0)$ is said to be *valid* because it is compatible with observing $\psi = ((2, 2, 0), (2, 2, 0))$ after simulating pooling (figure 1(a) and figure 2 (I)(1)), whereas $(2, 1, 0, 0)$ is not (Figure 1(b) and Figure 2 (II)). Therefore, the valid layouts for this set of genotypes are in proportion of $4/12 = 0.33$.

Similarly for any set of 4 genotypes and given ψ, only some permutations of this set will be valid data completions.

Last, we model as $(r, c) \in \{0, 1, 2\}^2$ the genotypes pair of two intersecting pools from ψ. With $\psi = ((2, 2, 0), (2, 2, 0))$, the samples in the block can intersect at $(r, c) = (0, 0)$, $(0, 1)$, $(1, 0)$, or $(1, 1)$. The latter case corresponds to the missing items. Using the aforementioned notations, the genotype of any sample in the block is fully parametrized by (ψ, r, c).

Formulation of the estimation problem for the missing data

For any pattern ψ in a pooling block, the objective is to estimate the most likely genotype distribution underlying any missing item $((r, c) = (1, 1))$ in $\mathbf{z}$ from the set of vectors $\mathbf{x}$ that are consistent with the pooling pattern observed. We denote this estimated distribution $\hat{\pi} := \overline{\hat{\pi}_i}$ with $\hat{\pi}_i$ computed for any block item i as

$$\hat{\pi}_i = (\hat{p_0}_i, \hat{p_1}_i, \hat{p_2}_i) \quad (3)$$
$$= (Pr(G = 0|\tilde{p_0}_i; \psi), Pr(G = 1|\tilde{p_1}_i; \psi), Pr(G = 2|\tilde{p_2}_i; \psi)) \quad (4)$$

where $\tilde{\pi}_i = (\tilde{p_0}_i, \tilde{p_1}_i, \tilde{p_2}_i)$ are prior estimates of the genotype distribution that z_i is sampled from. The mechanism defined in equation 4 is an inversion sampling of the priors $(\tilde{p_0}_i, \tilde{p_1}_i, \tilde{p_2}_i)$ with respect to ψ. The prior estimates can be initialized freely as long as they form a probability simplex.

Such a problem is usually solved with a Maximum Likelihood Estimation (MLE) approach or with methods based on Expectation-Maximization (EM). In the next sections, we propose likelihood estimation algorithms tailored for pooled genotype data that compute the iteration defined in equation 4.

Maximum Likelihood type II estimations (ML II)

Marginal Likelihood Maximization

In the ML II method, the likelihood of every valid layout is marginalized over the block parameters ψ, r, c. Therefore, the genotype distribution problem can be solved as a series of independent MLE for each set of block parameters. An illustrative example is provided for $\psi = ((2, 2, 0), (2, 2, 0))$ and $n_B = 16$ in Figure 2.

For any pooling pattern, the pooled data $\mathbf{z}$ is completed by enumerating all corresponding valid $\mathbf{x}$. The genotypes frequencies are evaluated from their expected counts in each $\mathbf{x}$ and the aggregated likelihood of the genotypes is maximized. The entire enumeration process is a variation to the classical MLE [3] where the distribution parameters are estimated from the proportions of data observed. The problem can also be formulated as a single-iteration Bayesian inference [2]. Finally, we obtain for $\psi = ((2, 2, 0), (2, 2, 0))$, $(r, c) = (1, 1)$ an estimate $\hat{\pi} = (0.214, 0.393, 0.393)$. In this model, we do not account for any prior information about the genotypes distribution, such that all genotypes are a priori equally likely when computing their frequencies in $\mathbf{x}$.

Maximum Marginal Likelihood with heterozygous degeneracy

By representing the genotypes with the random variable G introduced earlier, the two heterozygous states carrying alleles pairs $(0, 1)$ and $(1, 0)$ are merged into a single heterozygous genotype $G = 1$. This phenomenon can be defined as *heterozygotes degeneracy*. In other words, there are 2 equivalent micro layouts for each $G = 1$ enumerated, such that if n_1 is the number of heterozygotes in the pooling block, 2^{n_1} is the order of the degeneracy. To correct for this in a computationally efficient manner, the expected heterozygotes counts can be doubled before maximizing the likelihood of every genotype. With the previous example on Figure 2, the expected genotypes proportions become $(0.154, 0.564, 0.282)$. In compensation, when computing the final genotype frequencies, we downscale by a factor of 2 the arbitrary Eventually, this gives the same results as the previous calculation ($\hat{\pi} = (0.214, 0.393, 0.393)$).

Self-consistent Expectation-Maximization for estimating genotypes distributions

Let us assume we have prior beliefs about the genotypes distribution that we can include in the model presented for the ML II. For every sample in any observed pattern ψ, we seek to iteratively compute an estimate $\hat{\pi}$ of $\tilde{\pi}|\psi, r, c = (\tilde{p}_0, \tilde{p}_1, \tilde{p}_2)$ given an initial prior genotypes distribution $\tilde{\pi}^{(0)}$. We assume $\tilde{\pi}^{(0)}$ is not conditioned on ψ, which implies the hypothesis the likelihood of ψ is independent of the alternate allele frequency (AAF). This hypothesis is questionable as it is for example very unlikely to observe $\psi = ((4, 0, 0), (4, 0, 0))$ i.e. a pure $G = 0$ homozygous block, if the AAF at the pooled marker is close to 1. Therefore, we have also implemented other versions of our algorithm that introduce specific prior probabilities for the alleles with respect to ψ (unpublished work).

EM steps

Our algorithm starts every next iteration $m \geq 1$ as in the ML II by calculating the expectation of any valid layout $\mathbf{x}$, assuming $\tilde{\pi}^{(m-1)}$ are the true genotypes frequencies (E step). The expectation of $\mathbf{x}$ is computed as its relative likelihood

$$\mathbb{E}[\mathbf{x}|\mathbf{z};\tilde{\pi},\psi]^{(m)} = \frac{Pr(\mathbf{x}|\mathbf{z};\tilde{\pi},\psi)^{(m)}}{\sum\limits_{\mathbf{x}\subset\mathcal{X}} Pr(\mathbf{x}|\mathbf{z},\tilde{\pi},\psi)^{(m)}} \tag{5}$$

$$= \frac{Pr(\mathbf{z}|\mathbf{x})\,Pr(\mathbf{x};\tilde{\pi}^{(m-1)},\psi)}{\sum\limits_{\mathbf{x}\subset\mathcal{X}} Pr(\mathbf{z}|\mathbf{x})\,Pr(\mathbf{x};\tilde{\pi}^{(m-1)},\psi)} \tag{6}$$

$$\tag{7}$$

where $Pr(\mathbf{x};\tilde{\pi}^{(m-1)},\psi) \propto p_{\tilde{\pi}^{(m-1)}}$ and $Pr(\mathbf{z}|\mathbf{x}) = 0$ if $\mathbf{x} \notin \mathcal{X}_\psi$, else $Pr(\mathbf{z}|\mathbf{x}) = 1$ with

$$Pr(\mathbf{x};\tilde{\pi}^{(m-1)},\psi) = \frac{n!}{n_0!n_1!n_2!}\prod_{k=0}^{2}\left(\tilde{p}_k^{(m-1)}\right)^{n_k}, \quad \sum_{k=0}^{2} n_k = n_B, \tag{8}$$

n_k being the number of samples in $\mathbf{x}$ having the genotype k.

The mixing proportions from the E step are used in their turn to calculate the genotypes frequencies $\tilde{p_k}^{(m)},\ k \in \{0,1,2\}$ in $\mathbf{x}$ over all the data completions in $\mathcal{X}_\psi$ (M step) at iteration m as in a regular marginal likelihood maximization

$$\tilde{p_k}^{(m)} = \frac{\sum\limits_{\mathbf{x}\subset\mathcal{X}} n_k\,\mathbb{E}[\mathbf{x}|\mathbf{z},\tilde{\pi},\psi]^{(m)}}{\sum\limits_{k}\sum\limits_{\mathbf{x}\subset\mathcal{X}} n_k\,\mathbb{E}[\mathbf{x}|\mathbf{z};\tilde{\pi},\psi]^{(m)}}, \tag{9}$$

$$k \in \{0,1,2\} \tag{10}$$

where $\tilde{p_k}^{(m)}$ is the estimated frequency of genotype G_k at iteration m.

In the next step, we introduce rescaling weights $w = (w_0, w_1, w_2)$ as

$$\tilde{p}_k^{(m)\prime} = \frac{w_k\,\tilde{p}_k^{(m)}}{\tilde{p}_k^{(m-1)}},\ k \in \{0,1,2\} \tag{11}$$

$$\tag{12}$$

$$\tilde{p}_k^{(m)\prime\prime} = \frac{\tilde{p}_k^{(m)\prime}}{\sum\limits_k \tilde{p}_k^{(m)\prime}} \qquad \tilde{\pi}^{(m)} = (\tilde{p}_0^{(m)\prime\prime}, \tilde{p}_1^{(m)\prime\prime}, \tilde{p}_2^{(m)\prime\prime}) \tag{13}$$

These weights can be adjusted for the heterozygotes degeneracy to $(1,2,1)$, or set to $(1,1,1)$ if no correction is desired.

The algorithm repeats the E, M, and rescaling steps until an appropriate stopping criterion is reached.

At the last step, the posterior distribution of genotypes is calculated from the estimated genotypes frequencies with a downscaling transformation that compensates for the arbitrary w used in the previous iterations

$$\hat{p_k}^{(m)}|\psi = \frac{(1/w_k)\,\tilde{p}_k^{(m)}}{\sum\limits_k (1/w_k)\,\tilde{p}_k^{(m)}},\ k \in \{0,1,2\} \tag{14}$$

$$\hat{\pi}|\psi = (\hat{p_0}^{(m)}, \hat{p_1}^{(m)}, \hat{p_2}^{(m)}) \tag{15}$$

Estimation in a population under the assumption of uniformly distributed genotypes

A first hypothesis for determining the prior genotype probabilities to use models the genotypes as independent of each other with an uniform distribution $\tilde{\pi}^{(0)} = 0.33,\ k \in \{0,1,2\})$ at the iteration $m = 0$. In the same

example of the pattern $\psi = ((2,2,0),(2,2,0))$, we calculate hereafter the first EM steps ($m = 1$). In particular, for the valid layout $\mathbf{x} = (0,0,0,0,\ 2,0,0,0,\ 0,0,1,0,\ 0,0,0,0)$, the detailed formula of the E step is

$$\begin{aligned} Pr(\mathbf{x}) &= Pr(G_0)^2 * Pr(G_1)^1 * Pr(G_2)^1 \\ &= 0.33^2 * 0.33^1 * 0.33^1 \\ &= 0.33^4 \\ &= 0.012 \end{aligned}$$

and more generally for all valid permutations of $\mathbf{x} = (0,0,0,0,\ 2,0,0,0,\ 0,0,1,0,\ 0,0,0,0)$

$$\begin{aligned} Pr(\mathbf{x}; \tilde{\pi}^{(1)}, \psi) &= (4/12) * \frac{4!}{2!1!1!} * \tilde{p}_0^{(1)} * Pr(G_1)^1 * Pr(G_2)^1 \\ &= (4/12) * 12 * 0.33^2 * 0.33^1 * 0.33^1 \\ &= 4 * 0.33^4 \\ &= 0.048 \end{aligned}$$

We omit the terms for the 12 samples that have genotype 0 both in $\mathbf{x}$ and the mapped $\mathbf{z}$ ($(r,c) \neq 1$) since they are canceled out with the denominator later in the calculation of the mixing proportion of the valid layouts.

Overall for the 56 layouts $\mathbf{x}$ consistent with $\psi = ((2,2,0),(2,2,0))\}$,

$$\begin{aligned} \mathbb{E}[\mathbf{x}|\mathbf{z}; \tilde{\pi}, \psi]^{(1)} &= \frac{Pr(\mathbf{x}|\mathbf{z}; \tilde{\pi}, \psi)^{(1)}}{\sum_{\mathbf{x} \subset \mathcal{X}} Pr(\mathbf{x}|\mathbf{z}, \tilde{\pi}, \psi)^{(1)}} \\ &= \frac{4 * 0.33^4}{56 * 0.33^4} \text{ since the three genotypes } \{0,\ 1,\ 2\} \text{ are equally likely} \\ &= 0.0714 \end{aligned}$$

We note this quotient only depends on the number of valid layouts for each pattern.

At the M step, the mixing proportion $\mathbb{E}[\mathbf{x}|\mathbf{z}; \tilde{\pi}, \psi]^{(1)}$ serves as coefficient for maximizing the genotypes likelihood as their frequencies. For the valid permutations of $\mathbf{x} = (0,0,0,0,\ 2,0,0,0,\ 0,0,1,0,\ 0,0,0,0)$,

$$\begin{aligned} \tilde{p}_0(\mathbf{x})^{(1)} &= \mathbb{E}[\mathbf{x}|\mathbf{z}; \tilde{\pi}, \psi]^{(1)} * Pr(G = 0|\mathbf{x}) \\ &= 0.0714 * (2/4) \\ &= 0.0357 \\ \tilde{p}_1(\mathbf{x})^{(1)} &= 0.0714 * (1/4) \\ &= 0.0179 \\ \tilde{p}_2(\mathbf{x})^{(1)} &= 0.0714 * (1/4) \\ &= 0.0179 \end{aligned}$$

and overall, for all layouts layouts $\mathbf{x}$ consistent with $\psi = ((2,2,0),(2,2,0))\}$, we obtain

$$\begin{aligned} \tilde{p}_0^{(1)} &= 0.214 \\ \tilde{p}_1^{(1)} &= 0.393 \\ \tilde{p}_2^{(1)} &= 0.393 \end{aligned}$$

If we do not include heterozygotes degneracy in the model, we set $w = (1,1,1)$.

Hence, the updated prior distribution is

$$\begin{aligned} \tilde{p}_0^{(1)\prime\prime} &= 0.214 \\ \tilde{p}_1^{(1)\prime\prime} &= 0.393 \\ \tilde{p}_2^{(1)\prime\prime} &= 0.393 \end{aligned}$$

and the posterior after one iteration is equal to the prior

$$\hat{p}_0^{(1)} = 0.214$$
$$\hat{p}_1^{(1)} = 0.393$$
$$\hat{p}_2^{(1)} = 0.393$$

After one step, we actually obtain estimated genotype probabilities for the missing items that are MLE results: $\tilde{\pi}^{(1)} = (0.214, 0.393, 0.393)$. If continuing until 30 iterations, our algorithm converges and the final estimate of $\tilde{\pi}$ for $\psi = ((2,2,0),(2,2,0))$, $(r,c) = (1,1)$ is

$$\hat{p}_0^{(30)} = 0.170$$
$$\hat{p}_1^{(30)} = 0.415$$
$$\hat{p}_2^{(30)} = 0.415$$

Genotypes distribution in a population with uniform allelic dosage

The second approach assumes both alleles at the genotyped SNP are present in equal proportions in the population, hence the genotypes frequencies are $\tilde{\pi}^{(0)} = (0.25, 0.5, 0.25)$. As previously, we detail the case where $\psi = ((2,2,0),(2,2,0))$. The E step at iteration $m = 1$ becomes

$$\begin{aligned} Pr(\mathbf{x}; \tilde{\pi}^{(1)}, \psi) &= (4/12) * \frac{4!}{2!1!1!} * \tilde{p}_0^{(1)} * Pr(G_1)^1 * Pr(G_2)^1 \\ &= 4 * 0.25^2 * 0.5^1 * 0.25^1 \\ &= 0.0938 \end{aligned}$$

and

$$\mathbb{E}[\mathbf{x}|\mathbf{z}; \tilde{\pi}, \psi]^{(1)} = 0.0386$$

Consequently, at the M step, the genotypes frequencies from the set v in the mix are

$$\begin{aligned} \tilde{p}_0(\mathbf{x})^{(1)} &= \mathbb{E}[\mathbf{x}|\mathbf{z}; \tilde{\pi}, \psi]^{(1)} * Pr(G = 0|\mathbf{x}) \\ &= 0.0386 * (2/4) \\ &= 0.0193 \\ \tilde{p}_1(\mathbf{x})^{(1)} &= 0.0386 * (1/4) \\ &= 0.0097 \\ \tilde{p}_2(\mathbf{x})^{(1)} &= 0.0386 * (1/4) \\ &= 0.0097 \end{aligned}$$

and, overall layouts

$$\tilde{p}_0^{(1)} = 0.24$$
$$\tilde{p}_1^{(1)} = 0.38$$
$$\tilde{p}_2^{(1)} = 0.38$$

If our model accounts for the heterozygotes degeneracy, we set $w = (w_0, w_1, w_2) = (1, 2, 1)$.

Hence, after reweighing,

$$\tilde{p}_0^{(1)\prime} = 0.696$$
$$\tilde{p}_1^{(1)\prime} = 2.203$$
$$\tilde{p}_2^{(1)\prime} = 1.101$$

and after rescaling, the updated prior distribution is

$$\tilde{p}_0^{(1)''} = 0.174$$
$$\tilde{p}_1^{(1)''} = 0.551$$
$$\tilde{p}_2^{(1)''} = 0.275$$

Eventually, after 30 iterations, we obtain the estimated genotypes probabilities $\hat{\pi}$ for $\psi = ((2,2,0),(2,2,0))$, $(r,c) = (1,1)$

$$\hat{p}_0^{(30)} = 0.198$$
$$\hat{p}_1^{(30)} = 0.401$$
$$\hat{p}_2^{(30)} = 0.401$$

References

[1] A. Gordon et al. Y. Erlich K. Chang. “DNA Sudoku—harnessing high-throughput sequencing for multiplexed specimen analysis”. In: *Genome Research* 19 (2009), pp. 1243–1253.

[2] A. Tan, G. R. Abecasis, and H. M. Kang. “Unified representation of genetic variants”. In: *Bioinformatics* 31.13 (2015), pp. 2202–2204.

[3] C. B Do and S. Batzoglou. “”What is the expectation maximization algorithm?””. In: *Nature Biotechnology* 26.8 (2008), pp. 897–899.

Figures

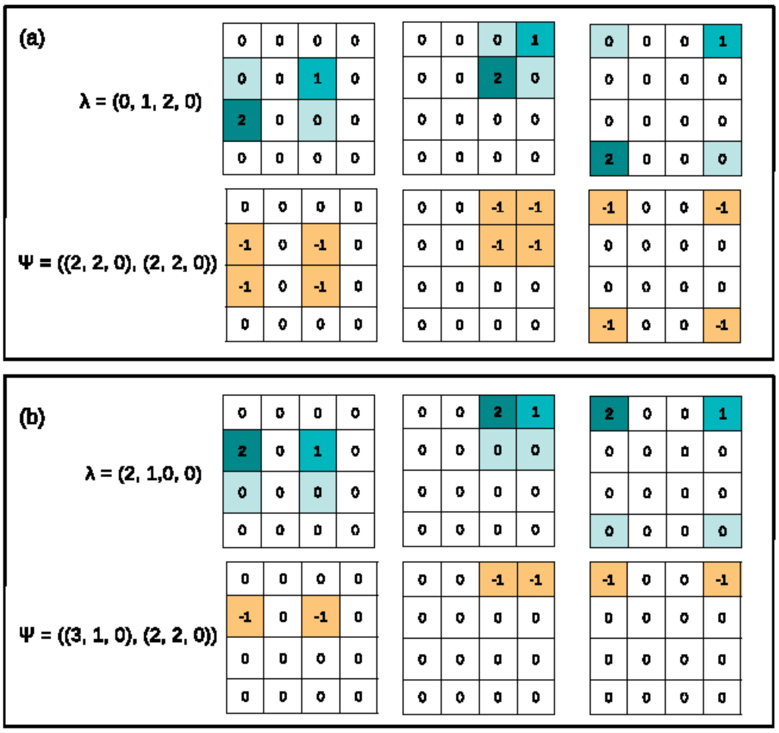

Figure 1: Examples of two pooling patterns obtained from two distinct permutations from the same set of genotypes. λ denotes the subvector of blue-colored genotypes that are possible completions of z. (a) The ALT-allele carriers are located on different rows and different columns, such that they never show up in the same pool. (b) The ALT-allele carriers are located on different columns but the same rows, such they are genotyped together in the row pool.

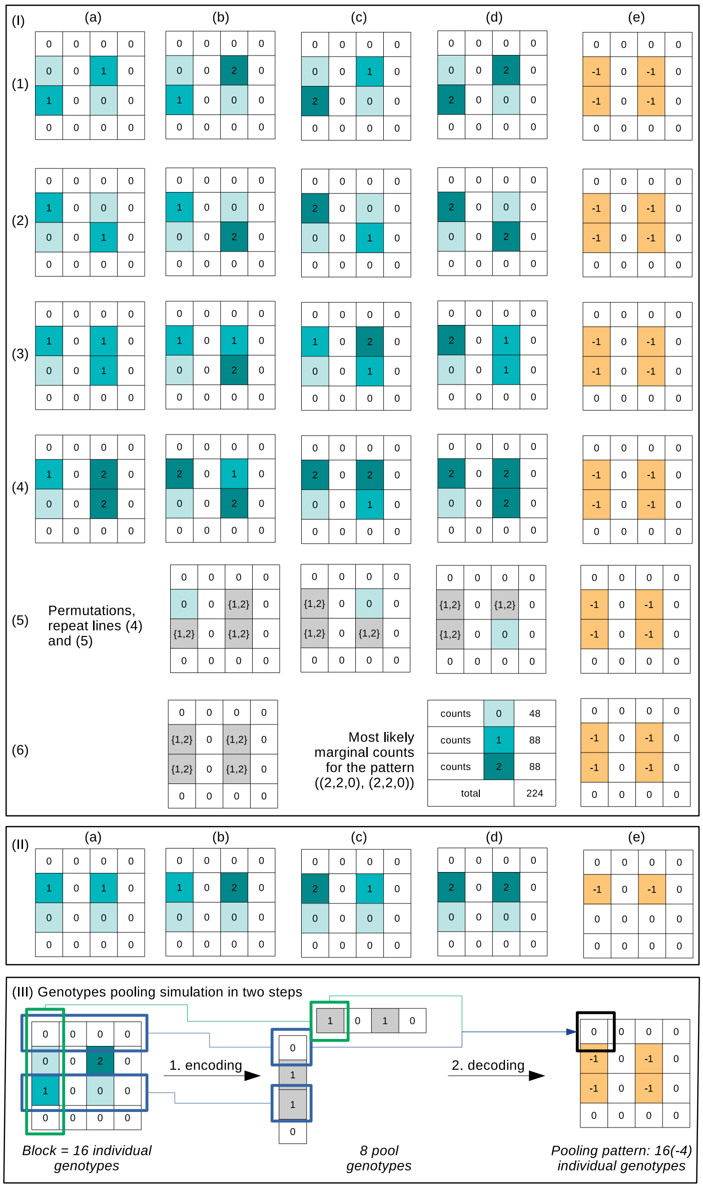

Figure 2: An enumerative method for completing ambiguous genotypes in pooling blocks
The figure shows the case of a block of 16 individuals with a pattern ψ of 4 undecoded items placed on two distinct pairs of row and column, while other items are decoded as REF-homozygotes ($\psi = ((2,2,0),(2,2,0))$). The pooling block is represented as square grid with 4 row-pools and 4 column-pools.
(I) Enumerating the valid layouts compatible with this pattern results in 56 outcomes. Over these combinations, the homozygous-REF genotype 0 (resp. heterozygous 1 and homozygous-ALT 2) appears 48 times (resp. 88 and 88), such that the estimated genotypes distribution fitted to the layout is $(0.214, 0.393, 0.393)$. This corresponds to a Maximum Marginal Likelihood estimation without heterozygotes degeneracy.
(II) For a given set of genotypes, some permutations result in layouts are not compatible with the observed pooling pattern ψ.
(III) Simulating pooling consists in a first encoding step which resolves the row- and column-pool's genotypes: 2 rows have genotype 0, 2 have genotype 1, none has genotype 2, and idem for the column-pools (noted as a pooling pattern $\psi = ((2,2,0),(2,2,0))$).The second step decodes the pooled data into individual genotypes.

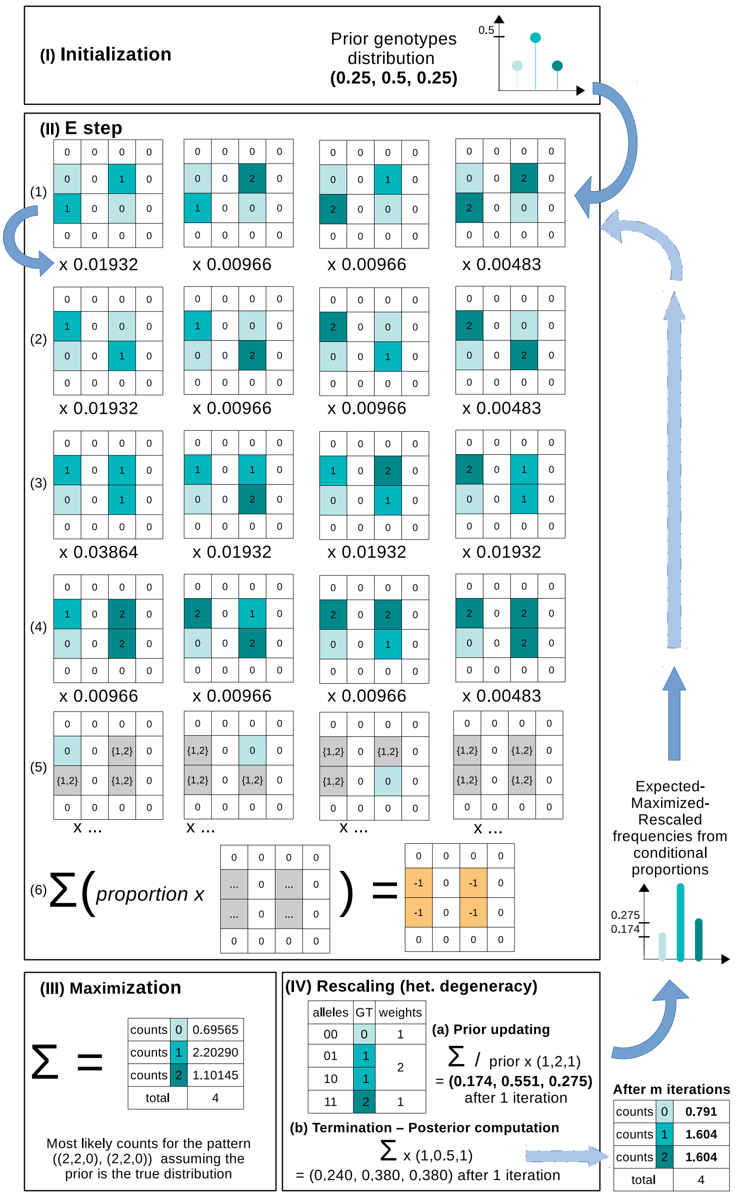

Figure 3: A self-consistent method for estimating ambiguous pooled genotypes with heterozygotes degeneracy The figure shows the case of a block of 16 individuals with a pattern ψ of 4 undecoded items placed on two distinct pairs of row and column, while other items are decoded as REF-homozygotes ($\psi = ((2,2,0),(2,2,0))$). The pooling block is represented as square grid with 4 row-pools and 4 column-pools.
(I) The distribution to estimate is initialized at a prior value that reflects a uniform alleles dosage at HWE.
(II) Data completing with valid layouts is performed similarly as in MML. For each combination, the prior distribution is used to compute its likelihood. We assume that the observed data (pooling pattern) is a continous mix of all valid layouts, which mixing proportions are proportional to their likelihood.
(III) The most likely genotypes counts given the prior distribution are derived from the marginal likelihoods weighted by their mixing proportions.
(IV) Rescaling is applied for accounting for heterozygotes degeneracy and layouts collapsing. Upscaling precedes prior updating, whereas downscaling occurs before computing the final MAP estimate of genotypes counts.
